# A Clinical and Laboratory Approach to the Evaluation of Innate Immunity in Pediatric CVID Patients

**DOI:** 10.3389/fimmu.2015.00145

**Published:** 2015-04-27

**Authors:** Necil Kutukculer, Elif Azarsiz, Neslihan Edeer Karaca, Ezgi Ulusoy, Guldane Koturoglu, Guzide Aksu

**Affiliations:** ^1^Department of Pediatric Immunology, Faculty of Medicine, Ege University, Izmir, Turkey

**Keywords:** innate immunity, CVID, primary immunodeficiency, NK cell function, test system

## Abstract

Defective adaptive immune responses are well studied in common variable immunodeficiency (CVID) patients; however, more focus is needed on innate immune system defects to explain CVID’s clinical and laboratory heterogeneity. This is the first study comparing migratory function of granulocytes, oxidative burst activity of phagocytic cells, surface integrin expressions on neutrophils and lymphocytes, natural killer (NK) cell numbers and cytotoxic activity, natural killer T cells, lymphocyte subsets such as CD8^+^CD28^+^, CD4^+^CTLA-4^+^ cells in CVID patients (*n*: 20) and healthy controls (*n*: 26). The relationship between laboratory findings and some clinical was also investigated. CD3^+^CD8^+^ T cytotoxic cells were found to be elevated in CVID patients, but CD3^+^CD8^+^CD28^+^ or CD3^+^CD8^+^CD28^−^ cells did not show any significant difference. CD4^+^CTLA-4^+^ cell percentages were significantly lower in CVID patients compared to healthy controls. Severe CVID patients had decreased percentages of NK cells with increased NK cell cytotoxicity suggesting possibly increased activation. Furthermore, CD3^−^CD16^+^CD56^+^CD28^+^ cells of CVID patients were elevated while percentage of CD28^−^ NK cells was decreased. Neutrophil migration percentages were lower but and oxidative burst activity was not affected. CD11a expressions on these cells were depressed in contrast to increased expression of CD18. Innate immunity defects may affect the extent of recurrence and severity of infections in CVID. Our observations highlight some of these associations and indicate the need for further similar studies for improving better innate system evaluation batteries for these patients. Further phenotypic correlations of these analyses will help clinicians reach a more definitive target for the molecular genetic diagnostic of pediatric CVID patients.

## Introduction

Common variable immunodeficiency (CVID) is a heterogeneous group of disorders characterized by hypogammaglobulinemia, defective specific-antibody production, increased susceptibility to infections of the respiratory and gastrointestinal tracts with encapsulated bacteria and a variety of complications such as autoimmunity, lymphoproliferation, splenomegaly, malignancy, and granulomas (1).

Although defective adaptive immune responses with impairment of T cell and B cell maturation and activation are common features in most CVID patients, less attention has been given to innate immune system defects as a possible explanation for CVID heterogeneity (2).

Neutrophils and monocytes are essential to innate immunity. They are the first cells to migrate from the blood to the inflammatory site where they kill pathogens and secrete various mediators that regulate innate and adaptive immunity (3). Functional and quantitative monocyte imbalances associated with T cell activation and with B cell disorders are observed in CVID (1). Monocytes can exhibit chronic hyperactivity and enhanced oxidative stress, which might contribute to autoimmune disorders, granuloma formation, and impaired natural killer (NK) cell-mediated cytotoxicity (1). Patients with AIDS showed a significant reduction of phagocytosis and respiratory burst which correlated with the number of CD4^+^ cells in comparison to asymptomatic HIV-infected individuals (4). Functional steps required for the activity of neutrophils and monocytes include migration (detected by Migratest), recognition, adhesion, phagocytosis, and killing of the target (detected by Phagoburst test) (3).

The role of endothelial adhesion molecules (selectins, CD62L, superimmunoglobulin family) in the pathogenesis of CVID and association of these molecules with the presence of splenomegaly has been reported previously (5). Integrins CD11a/CD18 (lymphocyte function-associated antigen-1/LFA-1), which are found on leukocytes help leukocyte transmigration to the inflammation area and are involved in a broad range of immunological processes including leukocyte extravasation, lymphocyte co-stimulatory signaling, and T-lymphocyte alloantigen-induced proliferation. To date, there is no study evaluating integrins on neutrophils and lymphocytes of CVID patients.

Natural killer cells as components of the innate defense system that targets and lyses tumor cells and virally infected cells have been shown to be significantly reduced in CVID (6). Furthermore, in a previous study, *in vitro* cytotoxicity of PBMC from CVID patients to the K562 cell line (presumed NK activity) have been found to be normal, suggesting a compensatory mechanism for the low numbers (7). CVID patients are prone to cancer development, particularly lymphoma, and the deficiencies in the functions or numbers of NK cells may contribute to heterogeneous infectious clinical findings, especially viral infections and tumor development in some cases.

Natural killer T (NKT) cells have been implicated in both up-regulation and down-regulation of numerous autoimmune and inflammatory conditions, as well as tumor/immune surveillance (8, 9). Autoimmunity and cancer are important components of CVID and the mechanisms in their development are yet poorly defined. Carvalho et al. (9) showed that NKT cells are circulating at the same frequency in the peripheral blood in CVID patients as healthy donors, but there is a skewing of NKT cell subsets in CVID patients.

CD28 and CTLA-4 both are co-stimulatory T cell molecules, which bind to antigen-presenting cells. CD28 transmits a stimulatory signal, whereas CTLA-4 transmits an inhibitory signal to T cells. It is shown that CD28 is extremely efficient at up-regulating IL-12-driven IFN-γ synthesis (which subsequently activates macrophage killing of organisms) by NK cells and Th1 polarization. CD28-deficient NK cells were shown to have markedly reduced ability to lyse tumor cells. CD28, by enhancing IFN-γ synthesis, may have a profound effect on innate immunity, Th1 development, and disease outcome (10, 11). The role of CD28-positive and CD28-deficient NK and NKT cells in the pathogenesis of CVID has not been studied. On the other hand, higher CTLA-4 expression, which competes for binding with CD28, is responsible for increased T cell self-reactivity. The mRNAs of CTLA-4 were reported to be expressed at lower levels in CVID patients compared to healthy controls (12).

In the present study, we evaluated the frequency and functional response of innate immune cells in order to elucidate the contribution of innate immunity to the pathogenesis or clinical heterogeneity of CVID. Clinical complications such as autoimmune diseases, gluten enteropathy, or granulomatous lesion formation were also compared with these parameters. Our main aim was to generate an innate functional array for these patients, search phenotypic associations to get clues for possible molecular genetic diagnosis of patients.

## Patients and Methods

Clinical and immunological data of 20 patients who fulfilled criteria for CVID from the out-patient and in-patient clinics of Ege University Faculty of Medicine, Department of Pediatric Immunology, Izmir, Turkey were evaluated.

Patients were diagnosed and classified according to both clinical and laboratory criteria reported by European Society for Immunodeficiencies/Pan-American Group for Immunodeficiency (ESID/PAGID) (13). Diagnosis criteria were as follows: (1) marked decrease of IgG (at least two SDs below the mean for age), (2) reduced serum IgA and/or IgM, (3) specific-antibody deficiency, (4) age >2 years, and (5) exclusion of other known causes of hypogammaglobulinemia. Ethics Committee approval and informed written consent for participation were obtained for all cases.

All demographic information including name, gender, date of birth, age at onset of symptoms, age at admission, age at diagnosis, family history and consanguinity, clinical symptoms or complications (autoimmune disease, chronic giardiasis, granulomatosis, lymphoma or any malignancy, lymphadenomegaly, splenomegaly, bronchiectasis, musculoskeletal system findings, celiac-like disease), follow-up duration, and laboratory data were recorded.

The patient group was evaluated as subgroups divided according to published disease severity criteria for CVID (14). Patients with splenomegaly and/or granulomatous diseases and/or bronchiectasis and/or lower baseline IgG values (at admission lower than 270 mg/dL) (*n*: 6) were included into the severe disease (SD) group. Patients diagnosed as CVID but who did not fulfill these criteria were grouped as moderate disease (MD) group (*n*: 14). As a control group, 26 healthy children (*n*: 26) within the similar age range were included.

All laboratory studies performed in patient and control group are listed in Table 1.

**Table 1:** **Flow cytometric analyses performed in patient and control groups**.

**Innate immunity**
Migration, phagocytosis, oxidative burst
Migratest (chemotaxis of granulocytes)*^flow cytometry^*
Phagoburst test*^flow cytometry^*
Integrins (adhesion molecules)
CD11a^+^ lymphocytes
CD11a^+^ neutrophils
CD18^+^ lymphocytes
CD18^+^ neutrophils
Natural killer cells
CD3^−^CD16^+^CD56^+^ (NK cell)
Natural killer cell cytotoxicity*^flow cytometry^*
CD3^−^CD16^+^CD56^+^28^+^
CD3^−^CD16^+^CD56^+^28^−^
Natural killer T cells
CD3^+^CD16^+^CD56^+^ (NKT cell)
CD3^+^CD16^+^CD56^+^28^+^
CD3^+^CD16^+^CD56^+^28^−^
CTLA-4^+^ cells
CD3^+^CD4^+^CTLA-4^+^

### Whole Blood Count Assay

Whole blood count, leukocyte counts, absolute neutrophil and lymphocyte counts, and relative ratio were performed with hemocounter Cell-Dyn 3700, Abbott Diagnostics, USA.

### Serum immunoglobulin assay

Serum immunoglobulins (IgG, IgA, IgM) were analyzed quantitatively by Dade Behring BNII nephelometer, Siemens, Germany and compared with age related normal levels (15).

### Flow cytometric analysis

All flow cytometric analyses were made using FacsCalibur and all cell subpopulations were acquired using CellQuest Pro software (BD Biosciences, USA) and Flow-Jo software version 8.8.9 (Treestar, USA).

The flow cytometric data in the text were given according to standardized publishing rules (16–18).

### Lymphocyte subsets assay

Percentages and absolute counts of lymphocyte subsets (CD3^+^, CD19^+^, CD3^+^CD4^+^, CD3^+^CD8^+^, CD3^+^HLA^−^DR^+^, CD3^−^CD16^+^CD56^+^, CD3^+^CD16^+^CD56^+^) were investigated by flow cytometry, FACSCalibur, Becton Dickinson (BD), USA. CD3/CD4/CD8/CD45, CD3/CD19/CD16/56/CD45, and CD3/HLA-DR multicolor antibody reagents were used for staining. Lymphocytes were gated based on their forward and side scatters and all cell subpopulations were acquired using Cell Quest-Pro software (BD Biosciences).

### CTLA-4 intracellular expressions on CD3^+^CD4^+^ lymphocytes

Heparinized whole blood (100 μL) was stained with anti-CD3 and anti-CD4 monoclonal antibodies (moAb) (Becton Dickinson, USA). Erythrocytes were incubated with 2 mL of lysing solution (Becton Dickinson) for 10 min. After washing with phosphate-buffered saline containing 0.1% bovine serum albumin (PBS-BSA), leukocytes were suspended in FACS permeabilizing solution (Becton Dickinson) for 10 min. The cells were stained with phycoerythrin (PE)-conjugated anti-CD152 (CTLA-4) moAb for 30 min, rinsed, and resuspended in PBS-BSA. The number of cells that were positive for intracellular CD4^+^ CTLA-4^+^ was expressed as a percentage of CD3^+^ gated cells using Flow-Jo software version 8.8.9 (Treestar, USA) (19, 20).

### CD28 surface expressions on CD3^−^CD16^+^CD56^+^ (NK), CD3^+^CD16^+^CD56 (NKT), and CD3^+^CD8^+^ (T cytotoxic) cells

Percentages of CD3^−^CD16^+^CD56^+^CD28^+^, CD3^+^CD16^+^CD56 CD28^+^, *CD3*^+^*CD8*^+^CD28^+^, *CD3*^+^*CD8*^+^CD28^−^ subpopulations were analyzed according to cell surface staining instructions using anti-CD3 APC, anti-CD8 PE, anti-CD16 PE, anti-CD56 PE, and anti-CD28 FITC moAb (Becton Dickinson, USA) in heparinized blood samples. CD28 percentages on gated NK and NKT cells were analyzed with Flow-Jo software version 8.8.9 (Treestar, USA). T cytotoxic cells were given as *CD8*^+^CD28^+^ percentages on CD3^+^ gated cells.

### CD11a and CD18 surface expressions on neutrophil granulocytes and lymphocytes

Surface expressions of CD11a and CD18 on lymphocytes and neutrophil granulocytes were analyzed according to cell surface staining instructions using anti-CD11a FITC and anti-CD18 PE moAb (Becton Dickinson, USA) in heparinized blood samples. Gating strategy for defining the integrins was as follows: lymphocytes and neutrophils were gated based on their forward and side scatters. Compensations were made if needed and the percentages of the integrins were assessed using Cell Quest-Pro software.

### Oxidative burst activity of monocytes and granulocytes (phagoburst test)

The quantification of the oxidative burst activity of monocytes and granulocytes in heparinized whole blood was determined by Phagoburst test, Orpegen Pharma, Heidelberg, Germany. The test determines the percentage of phagocytic cells, which produce reactive oxidants in the presence of stimulants as *Escherichia coli* (*E. coli*), fMLP (*n*-formyl-methionine-leucine-phenylalanine), and PMA (phorbol-12-myristate-13-acetate) and cells’ enzymatic activity. The kit has been validated using an independent assay in preliminary studies and the results were similar (Normal reference values are as follows: for monocytes by stimulant *E. coli*: 70–100%; for granulocytes by stimulant *E. coli*: 95–100%, fMLP: 1–20%, and PMA: 99–100%).

### Chemotactic function of granulocytes(migratest)

Chemotactic function of neutrophilic granulocytes was evaluated by Migratest, Orpegen Pharma, Heidelberg, Germany. The test allows determination of the number of neutrophils, which have migrated through cell culture inserts toward a concentration gradient of the chemoattractant fMLP. More than 95% activated granulocytes were accepted as normal.

### Cytotoxic activity of NK cells

Quantification of the cytotoxic activity of NK cells was made according to NK test instructions, Orpegen Pharma, Heidelberg, Germany. K562 (chronic myeloid leukemic cell line) target cells were cultured and labeled with a lipophilic green fluorescent membrane dye, DIOC_18_ (3) (3,3′-dioctadecyloxa carbocyanine perchlorate), Life Technologies, USA, and then incubated with effector mononuclear cells, which were obtained from fresh heparinized blood by density gradient centrifugation on Biocoll (Biochrom GmbH) separating solution. The percentages of target cells killed by effector cells and live cells were determined. The percent specific cytotoxicity of mononuclear cells (presumed NK activity) is determined by subtracting the percentage of dead cells in the incubated targets alone (target negative control tube) from the percentage of killed target cells in the test samples (test tube). Normal reference percent specific toxicity differs according to target/effector cell ratio. All assays were performed in 1:25 target/effector cell ratio and a tube with 1:25 target/effector cell ratio including interleukin-2 was used as positive control tube. All cell subpopulations were measured by flow cytometry and analyzed with CellQuest Pro software (BD Biosciences).

### Specific-antibody response

Specific-antibody responses to tetanus and *Haemophilus influenza* antigens were analyzed by commercial ELISA kits and were previously recorded.

### Evaluation of autoimmunity

Antinuclear antibody (ANA) positivity in serum was determined by immunoflorescence (IF) on mosaic Hep-20-10/liver monkey cell slides (Euroimmun, Lübeck, Germany) in a double-blind setting, in order to evaluate autoimmunity in patients. ANA IF titers of 1:100 were taken as cut-off titers. Anti-neutrophil cytoplasmic antibody (ANCA) positivity with a 1:16 cut-off titer was also evaluated by IF. Titrimetric qualitative determinations (±) were used. All autoimmune diseases (autoimmune hemolytic anemia, autoimmune thrombocytopenia, rheumatoid arthritis, pernicious anemia) records were also evaluated.

### Evaluation of celiac-like disease findings

Gluten enteropathy was evaluated by patients’ data records for anti-gliadin (AGA) and anti-tissue transglutaminase (TTG) IgA antibodies levels, which were performed with ELISA.

### Statistics

All clinical and laboratory data were evaluated in relation with each other and compared in different patient profiles and control groups. Statistical analyses were performed by using SPSS Windows Version 17.0, SPSS Inc., Chicago, IL, USA. Data were expressed as mean plus or minus SD except when indicated otherwise. Correlation comparisons between paired samples were made by Pearson’s product moment correlation coefficient. Statistical comparisons of numeric data were made using Student’s *t*-test and classified data were evaluated by chi-square test. A two-sided *p*-value <0.05 was considered to indicate statistical significance.

## Results

A total of 20 CVID patients (*n* = 3 female, 15% and *n* = 17 male, 85%) with a mean age of 173.2 ± 77.9 months and 26 healthy controls (*n* = 12 female, 46% and *n* = 14 male, 54%) with a mean age of 133.09 ± 63.0 months were included in this study.

All demographic and clinical characteristics of the patient group are summarized in Table 2. According to disease severity criteria, 30% of patients (*n* = 6) had severe and 70% of patients (*n* = 14) had MD. In whole patient group, the mean age at the onset of symptoms was 70.0 ± 60.8 and 93.7 ± 58.7 months at diagnosis. A total of 17% of the patients (*n* = 3) were born to consanguineous parents. Consanguinity had a significant relationship with disease severity (*p*: 0.052). Family history for primary immune deficiencies was positive in five patients (25%). Infection was the most common cause of admission for 18 patients. The other reasons for admission were autoimmune hemolytic anemia and severe lymphadenomegaly. The most frequent infection types were sinopulmonary tract infections and gastroenteritis. Specific-antibody response was negative in six patients (30%). All patients were followed up for 80.6 ± 70.3 months. IVIg replacement therapy was started at the mean age of 83 ± 51.1 months and the mean duration for IVIg replacement was 111.5 ± 59.9 months. Neither the mean age of starting IVIg therapy (*p*: 0.200) nor IVIg duration (*p*: 0.394) showed statistical significance in relation to disease severity.

**Table 2:** Patient group characteristics (mean ± SD) (*p < 0.05, independent *t*-test).

	All patients (*n*: 20)	Moderate disease (*n*: 6)	Severe disease (*n*: 14)	*p*	Male (*n*: 17)	Female (*n*: 3)	*p*
Age at the time of study (months)	173.2 ± 77.9	163.2 ± 63.1	199.4 ± 112.6	0.394	184.2 ± 76.1	118.6 ± 75.6	0.192
Age at onset of symptoms (months)	70.0 ± 60.8	58.3 ± 55.7	108 ± 69.2	0.257	71 ± 62.1	65.6 ± 67	0.908
Age at diagnosis (months)	93.7 ± 58.7	84.6 ± 58.3	123 ± 57.4	0.297	96.2 ± 61.2	81.6 ± 53.8	0.709
Follow-up duration (months)	80.6 ± 70.3	79.3 ± 67.5	84.7 ± 89.9	0.899	92.7 ± 71.9	24 ± 10.4	0.004*
IVIg treatment duration (months)	111.5 ± 59.9	94.7 ± 43.2	178.5 ± 89.8	0.404	111.5 ± 59.9

Major complications in the patient group, observed during follow-up were as follows: bronchiectasis (*n* = 4, 20%), splenomegaly (*n* = 5, 25%), osteoporosis (*n* = 6, 30%), delayed growth (*n* = 6, 30%), autoimmunity (*n* = 5, 25%), hepatic enlargement (*n* = 4, 20%), gluten-like enteropathy (*n* = 2, 10%), granuloma formation (*n* = 4, 20%), and lymphoma (*n* = 2, 10%). Failure to thrive, autoimmunity, gluten-like enteropathy, osteoporosis, bronchiectasis, splenomegaly, and hepatomegaly were the significantly increased complications in SD group. The disease severity showed no significant correlation with granuloma formation (*p*: 0.243), lymphoma evolution (*p*: 0.360), or autoimmunity (*p*: 0.360).

Autoimmune clinical findings were observed in five patients; chronic arthritis (*n* = 1), inflammatory bowel disease (*n* = 2), relapsing polychondritis (*n* = 1), autoimmune hemolytic anemia (*n* = 1), and thrombocytopenia (*n* = 2), spondylarthropathy of the hip (*n* = 1), sacroileitis (*n* = 1), and vasculitis (*n* = 1).

General laboratory data of study group are summarized in Table 3. At the time of diagnosis, white blood cell (WBC) and absolute lymphocyte counts did not show any significant difference between disease and control groups.

**Table 3:** Laboratory data of CVID patient and control groups (*p*^1^: between moderate and severe disease groups; *p*^2^: between patient and control groups) (*p < 0.05).

		Patient group (mean ± SD)				Control group (mean ± SD)	*p*^2^
		All patients (*n*: 20)	Moderate disease	Severe disease	*p*^1^	
Leukocyte count	(10^3^/μL)	7.47 ± 4.39	7.52 ± 4.94	7.27 ± 1.83	0.922	7.21 ± 2.28	0.817
Absolute lymphocyte	cells/mm^3^	2642.7 ± 1542.1	2686.6 ± 1690.9	2488.8 ± 1023.1	0.829	2728.1 ± 1024.0	0.840
Initial IgG	(mg/dL)	405.8 ± 181.6	461.7 ± 162.3	210.2 ± 85.1	0.010*	–	–
Initial IgA	(mg/dL)	43.4 ± 60.3	35.7 ± 45.6	68.4 ± 100	0.361	–	–
Initial IgM	(mg/dL)	46.3 ± 41.6	54.3 ± 44.1	18.1 ± 4.38	0.129	–	–
CD19^+^ B cells	%	7.62 ± 6.47	8.92 ± 7.27	4.60 ± 2.41	0.179	13 ± 5.53	0.004*
	cells/mm^3^	260.8 ± 393.2	289.5 ± 437.3	160.2 ± 173.7	0.578	372.6 ± 215.7	0.269
CD3^+^ T cells	%	78 ± 11.2	74.3 ± 10.8	86.6 ± 6.46	0.019*	71.7 ± 8.90	0.039*
	cells/mm^3^	1890.2 ± 1177.1	1688 ± 1012.6	2598.2 ± 1596.6	0.180	1875.3 ± 747.9	0.963
CD3^+^4^+^ T helper cells	%	37.4 ± 12.6	37.8 ± 13.2	36.5 ± 12.1	0.831	39.7 ± 9.12	0.476
	cells/mm^3^	914.1 ± 651.3	843.5 ± 534.4	1161.2 ± 1030.4	0.406	1012.8 ± 426.1	0.574
CD3^+^CD8^+^ T cytotoxic cells	%	35.6 ± 13	31.9 ± 9.80	44.2 ± 16.5	0.052*	26.2 ± 6.28	0.007*
	cells/mm^3^	840.4 ± 610.5	756 ± 578.9	1135.7 ± 714.3	0.286	683.9 ± 268.4	0.324
CD3^+^CD8^+^CD28^+^	%	22.2 ± 8.19	22.1 ± 8.38	22.4 ± 8.53	0.952	23 ± 6.18	0.732
	cells/mm^3^	0 ± 0	0 ± 0	0 ± 0	–	149.3 ± 52.9	0.313
CD3^+^CD8^+^CD28^−^	%	16.1 ± 11.6	14.9 ± 9.22	18.7 ± 16.4	0.523	15.1 ± 7.60	0.634
	cells/mm^3^	0 ± 0	0 ± 0	0 ± 0	–	102.8 ± 79.2	0.112
CD3^+^HLA-DR^+^ Active T cells	%	17.9 ± 18	13.2 ± 11.6	28.7 ± 26.1	0.216	6.18 ± 3.11	0.009*
	cells/mm^3^	378.8 ± 615.3	215.5 ± 253	950.2 ± 1143.9	0.290	107.7 ± 74.5	0.080
CD4^+^CTLA-4^+^ cells on CD3^+^	%	0.47 ± 0.47	0.39 ± 0.42	0.68 ± 0.61	0.319	1.36 ± 1.50	**0.016**
	cells/mm^3^	7.78 ± 8.60	6.82 ± 6.76	11.3 ± 15.1	0.661	26.3 ± 35.2	0.065

### Immunoglobulins

Baseline IgG, IgM, and IgA levels in the patient group were 405.8 ± 181.6, 46.3 ± 41.6, and 43.4 ± 60.3 mg/dL, respectively, before commencement of IVIg therapy. There was a significant difference between severe and MD groups (*p*: 0.010) for IgG concentrations, which were lower in severe group (Table 3).

### Lymphocyte subsets

Percentages of CD19^+^ B cells were lower in the patient group compared to controls (*p*: 0.004). The decrease was more significant in the SD group (Table 3), but there was no significant difference correlating to disease severity. A statistically significant difference between the two groups for CD3^+^ T cells (*p*: 0.039) was relative to decrease in CD19^+^ B cells. CD3^+^CD8^+^ T cytotoxic cells and HLA-DR^+^ active T cells were higher in the SD group than the MD group and there was also a significant difference between patient and control groups, respectively (*p*: 0.007, *p*: 0.009). CD3^+^CD8^+^CD28^+^ (*p*: 0.732) or CD3^+^CD8^+^CD28^−^ cells (*p*: 0.634) (Table 3).

### CTLA-4 cells

CD4^+^CTLA-4^+^ cell percentages on CD3^+^ lymphocytes were 0.47 ± 0.47 and 1.36 ± 1.50 in disease and control groups and there was significant difference between both groups (*p*: 0.016). In the SD group, it was higher (0.68 ± 0.61) than in the MD group (0.39 ± 0.42), but the difference here was not significant (*p*: 0.319).

### NK cells and NK cell subsets

Percentages of CD3^−^CD16^+^CD56^+^ natural killer cells of patient and control groups did not show any statistically significant difference (*p*: 0.107), but it was significantly lower in the SD group than in the moderate group (*p*: 0.012). On the CD3^−^CD16^+^CD56^+^ cells, the percentages of CD28 positivity were higher (*p*: 0.008) and CD28 negativity were lower in the patient group than the control group (*p*: 0.006) (Table 4). Ratio of NK cells (*p*: 0.401), CD28^+^ NK (*p*: 0.915), or CD28^−^ NK (*p*: 0.944) cells did not effect the presence of lymphoma.

**Table 4:** Natural killer cells (NK cell) in CVID patient and control groups (*p*^1^: between moderate and severe disease groups; *p*^2^: between patient and control groups) (*p < 0.05).

		Patient group (mean ± SD)				Control group (mean ± SD)	*p*^2^
		All patients	Moderate disease	Severe disease	*p*^1^	
CD3^−^CD16^+^CD56^+^	%	20.5 ± 18.9	25.6 ± 20.4	8.83 ± 5.69	0.012*	26.2 ± 11.6	0.107
	cells/mm^3^	103.7 ± 154.8	126.5 ± 169.5	23.8 ± 18.2	0.253	232.4 ± 256.7	0.073
CD3^−^CD16^+^CD56^+^28^+^	%	20.0 ± 30.3	17.4 ± 29.6	26.2 ± 33.8	0.570	0.17 ± 0.27	0.008*
	cells/mm^3^	22.6 ± 62	25.2 ± 70.2	13.5 ± 18.2	0.752	0.39 ± 0.59	0.147
CD3^−^CD16^+^CD56^+^28^−^	%	78.3 ± 31.1	81.6 ± 30.7	70.8 ± 33.7	0.492	99.8 ± 0.26	0.006*
	cells/mm^3^	80.1 ± 148.29	100.3 ± 163.6	9.45 ± 3.740	0.293	232.0 ± 256.2	0.034*

### NK cell cytotoxicity

Effector cells, which were collected from PBMC of CVID patients and healthy controls, were incubated with DIOC dyed K562 target cells and then live and dead cell groups were determined using flow cytometry. Increased percentages of dead target cells in positive control tubes in comparison with negative control and test tubes showed us that the test was trustworthy. The percentage of dead target K562 cell was significantly higher in the patient group than the control group (*p* < 0.0001). SD group patients had higher dead cell percentages than MD group, but the difference was not statistically significant (*p*: 0.075).

**Figure 1:**
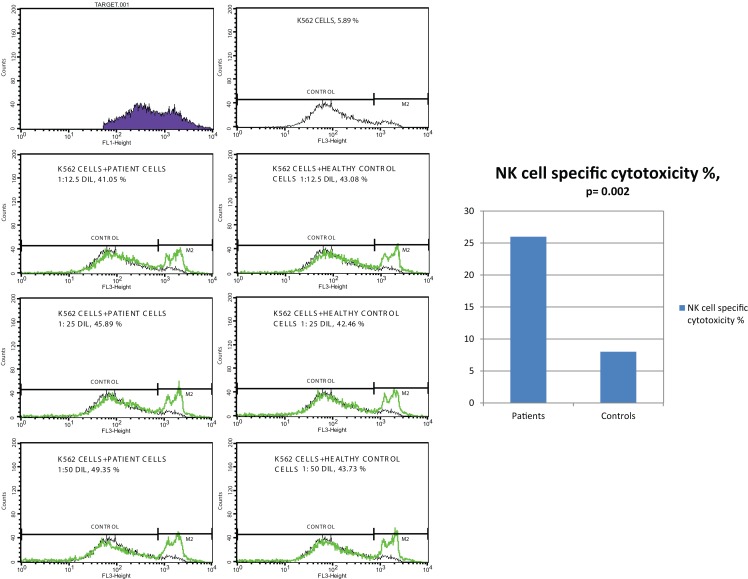
**NK cell specific toxicity assay, comparison of patients with controls (*p* = 0.002)**.

*In vitro* cytotoxicity of PBMC (presumed NK activity) from CVID patients to the target K562 cell line was determined by subtracting the percentage of dead cells in the incubated targets alone (target negative control tube) from the percentage of killed target cells in the test samples (test tube). The percentage of NK cell specific toxicity has been found to be higher in the patient group than the control group (*p*: 0.002) but the difference was not statistically significant between moderate and SD groups (0.873) (Table 5) (Figure 1).

**Table 5:** The mean percentage of target K562 cells killed by effector NK cells (at 1:25 target/effector cell ratio) and NK cell specific toxicity in patient and control groups (*p*^1^: between moderate and severe disease groups; *p*^2^: between patient and control groups) (*p < 0.05).

		Patient group (mean ± SD) (*n* = 11)				Control group (mean ± SD) (*n* = 14)	*p*^2^
		All patients	Moderate disease	Severe disease	*p*^1^	
Target dead cells%	Test tube (1:25)	34.1 ± 11.4	30.4 ± 9.7	44.0 ± 10.9	0.075	10.8 ± 14.6	0.000*
	Positive control tube (1:25-IL2)	35.7 ± 11.7	32.4 ± 10.2	44.6 ± 12.7	0.129	11.8 ± 15.5	0.000*
	Negative control tube	8.16 ± 16.0	4.1 ± 3.91	18.8 ± 31.5	0.189	5.71 ± 12.9	0.676
NK cell specific toxicity%		26.9 ± 13.3	27.3 ± 11.6	25.8 ± 27.3	0.873	8.28 ± 10.6	0.002*

### NKT cells

CD3^+^CD16^+^CD56^+^ cell percentages did not show any difference between patient and control groups (*p*: 0.288) or between severe and moderate groups (*p*: 0.077). Neither CD28^+^ nor CD28^−^ cell percentages on NKT cells showed difference between the two groups (Table 6). NKT cells (*p*: 0.256), CD28^+^ NKT (*p*: 0.592), or CD28^−^ NKT (*p*: 0.268) cells did not show any difference in the presence of lymphoma or autoimmunity.

**Table 6:** Natural killer T cells (NKT cell) in CVID patient and control groups (*p*^1^: between moderate and severe disease groups; *p*^2^: between patient and control groups) (*p < 0.05).

		Patient group (mean + SD)				Control group (mean + SD)	*p*^2^
		All patients	Moderate disease	Severe disease	*p*^1^	
CD3^+^CD16^+^CD56^+^	%	0.57 ± 0.49	0.70 ± 0.49	0.27 ± 0.37	0.077	1.51 ± 3.85	0.288
	cells/mm^3^	0 ± 0	0 ± 0	0 ± 0	–	19.5 ± 24.4	–
CD3^+^CD16^+^CD56^+^28^+^	%	15.6 ± 19.0	18.7 ± 20.6	8.55 ± 13.4	0.287	19.0 ± 17.5	0.544
	cells/mm^3^	0 ± 0	0 ± 0	0 ± 0	–	2.53 ± 3.01	–
CD3^+^CD16^+^CD56^+^28^−^	%	67.3 ± 3.80	63.4 ± 32.1	76.4 ± 31.9	0.417	78.6 ± 17.5	0.164
	cells/mm^3^	0 ± 0	0 ± 0	0 ± 0	–	16.6 ± 23.3	–

### Migration

Four patients in the disease and one patient in the control group showed decreased migration capacity if more than 95% activated granulocytes were accepted as normal when the number of neutrophils that have migrated through cell culture inserts toward a concentration gradient of the chemoattractant fMLP were determined. Percentage of activated granulocytes was 93.7 ± 5.08 and 97.7 ± 4.98 in disease and control groups, respectively (*p*: 0.025) (Table 7).

**Table 7:** Migration and oxidative burst activity in CVID patient and control groups (*p*^1^: between moderate and severe disease groups; *p*^2^: between patient and control groups) (*p < 0.05).

		Patient group (mean ± SD)				Control group (mean ± SD)	*p*^2^
		All patients	Moderate disease	Severe disease	*p*^1^	
Migratest (% activated granulocytes)		93.7 ± 5.08	92.5 ± 5.55	96.4 ± 2.23	0.037*	97.7 ± 4.98	0.025*
Oxidative burst (% phagocytic cells which produce reactive oxidants)	fMLP	6.55 ± 5.48	6.11 ± 5.53	7.57 ± 5.72	0.600	6.95 ± 6.54	0.836
	PMA	94.9 ± 7.86	94.5 ± 9.34	95.9 ± 2.57	0.728	98.1 ± 3.19	0.109
	*E. coli*	91.8 ± 13.7	90.7 ± 15.8	94.1 ± 7.33	0.625	95.5 ± 4.43	0.257

### Phagocytosis

Percentages of phagocytic granulocytes, which produce reactive oxidants in the presence of stimulants as fmLP, PMA, and *E. coli* were 6.55 ± 5.48, 94.9 ± 7.86, and 91.8 ± 13.7 in patients and 6.95 ± 6.54, 98.1 ± 3.19, and 95.5 ± 4.43 in the control group, respectively. Oxidative burst activity was found to be decreased in six patients (30%); however, the groups did not show any significant difference (Table 7).

### CD11a^+^ and CD18^+^ cells

**Figure 2:**
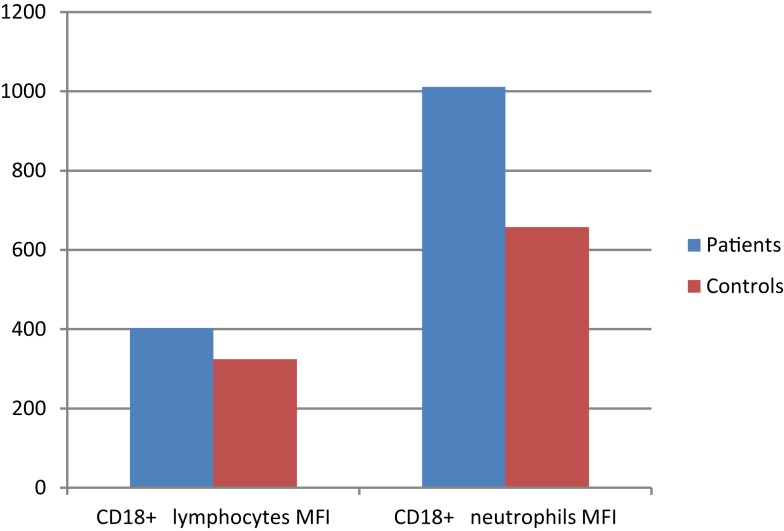
**CD18 surface expression (MFI) on leukocytes of CVID patients compared with controls (*p* < 0.05)**.

Integrin CD11a percentages on lymphocytes and neutrophils showed significant difference between patient and control groups (*p*: 0.042, *p*: 0.024, respectively) while CD18 percentages did not differ in either group (*p*: 0.346, *p*: 0.935), but mean florescence intensity (MFI) of CD18 on both neutrophils and lymphocytes was significantly increased in the patient group (*p*: 0.012 and *p*: 0.035, respectively) (Table 8) (Figure 2).

**Table 8:** Integrins (adhesion molecules) in CVID patient and control groups (*p*^1^: between moderate and severe disease groups; *p*^2^: between patient and control groups) (*p < 0.05).

		Patient group (mean ± SD)				Control group (mean ± SD)	*p*^2^
		All patients	Moderate disease	Severe disease	*p*^1^	
CD11a^+^ lymphocytes	%	91.6 ± 10.2	89.7 ± 11.8	95.7 ± 3.18	0.249	96.6 ± 5.30	0.042*
	cells/mm^3^	2227.7 ± 1352.3	2018.8 ± 1177.7	2906.5 ± 1844.4	0.264	2549.5 ± 1031.4	0.418
CD11a^+^ neutrophils	%	78.4 ± 29.5	78.5 ± 32.6	78.3 ± 23.2	0.988	95.1 ± 9.52	0.024*
CD18^+^ lymphocytes	%	96.6 ± 6.28	96.2 ± 7.58	97.5 ± 1.68	0.691	98.3 ± 5.45	0.346
	cells/mm^3^	2428.1 ± 1534	2241 ± 1410.1	3036.2 ± 1987.8	0.382	2643.4 ± 1073.4	0.626
	MFI	401.9 ± 112.4	382.9 ± 90.0	446.3 ± 153.6	0.564	323.5 ± 106.6	0.035
CD18^+^ neutrophils	%	99.6 ± 0.59	99.8 ± 0.04	99.2 ± 1.0	0.196	99.7 ± 0.55	0.935
	MFI	1010.5 ± 597.8	865 ± 448.1	1349.8 ± 798.9	0.322	657.2 ± 401.4	0.012

## Discussion

Common variable immunodeficiency is a highly heterogeneous immunodeficiency syndrome and about 20% of CVID patients have a family history of immune deficiency and parental consanguinity. In our study, mean age at the beginning of symptoms was about 7 years and mean age at diagnosis was about 8 years and parental consanguinity was 17%. Consanguinity had significant relationship with disease severity. Although diagnostic delay was not long (about 1 year), 20% of patients presented with bronchiectasis and 30% with osteoporosis and growth failure.

Some CVID patients may present low peripheral B cell numbers associated with defective terminal B cell maturation. Wehr et al. reported that most of their CVID patients had low B cell counts (21). Similar to our previous study (22), there was no significant difference between severe and moderate patient groups in terms of B cell numbers; however, B cells in total CVID patients were significantly lower than in healthy controls (Table 3). CD3^+^CD8^+^ T cytotoxic cells and HLA-DR^+^ active T cells were higher in SD group than MD group and there was a significant difference between patient and control groups (*p*: 0.007, *p*: 0.009, respectively).

In a study conducted by North et al. (23), in CVID patients, there was a significant increase in the mean proportion of CD3^+^CD8^+^CD28^−^cells compared with normal donors, but normal proportions of HLA^−^DR^+^CD8^+^ cells. The authors also reported a failure of activation and IFN-γ production in the suppressor CD28^−^ population and concluded that this phenotypic and functional shift in the CD8^+^ cells may lead to the failure of CD4^+^ T cells to respond to antigen and be causing the antibody deficiency seen in this disease (23). In contrast to these findings, in our study, CD3^+^CD8^+^CD28^+^ (cytotoxic) (*p*: 0.732) or CD3^+^CD8^+^CD28^−^ (suppressor) cells (*p*: 0.634) did not had any statistically significant difference between the two groups (Table 3).

Autoimmune disorders occur with a higher incidence in CVID patients than in the general population. Approximately 20–30% of CVID patients develop autoimmune disorders, sometimes present as the first manifestation of the disease (24). Juvenile idiopathic arthritis (JIA), pernicious anemia, autoimmune tiroiditis, alopecia areata, primary biliary cirrhosis, vitiligo, and systemic lupus erythematosus have been described in CVID; however, the most common types reported are idiopathic thrombocytopenic purpura (ITP) and autoimmune hemolytic anemia (AIHA) (24). AIHA (4/14, 28.5% of total autoimmune diseases observed in the study), JIA (4/14, 28.5%), and ITP (3/14, 21.4%) were the most common autoimmune disorders in Abolhassani et al.’s study (24). In our study, autoimmune diseases were observed in five cases (25%) and four of them were in the SD group.

CTLA-4 is a co-stimulatory molecule, which transmits inhibitory signals to T cells. In our previous study (22), we have reported that carrying G allele or GG or AG genotypes increases the risk of developing CVID approximately twofold. Intracellular CTLA-4 expressions were examined by flow cytometry in this study. CD4^+^CTLA-4^+^ percentages on CD3^+^ lymphocytes were 0.47 ± 0.47 and 1.36 ± 1.50 in disease and control groups and there was significant decrease in CVID patients. Ueda et al. (25) have reported that certain common autoimmune diseases are caused in part by inherited changes in CTLA-4 expression that presumably increase T cell self-reactivity. Schubert et al. (26) identified nonsense, splice site, and missense mutations in CTLA-4 genes in 14 patients from six families. These patients presented with a complex immune dysregulation syndrome characterized by hypogammaglobulinemia, recurrent infections, and multiple autoimmune clinical features diagnosed as CVID (26). However, in our study, CTLA-4 expressions in our five CVID cases with autoimmunity did not show any significant difference from other CVID cases and healthy donors (data not shown).

Trujillo et al. (2) found a marked, though not significant, reduction in absolute numbers of NK cells in CVID patients. Circulating NK cell numbers are clearly depleted in CVID patients in most of the studies; this appears to have no short-term clinical relevance, since the patients are not prone to herpes viruses, as has been reported in some patients with isolated NK cell deficiencies (6). In our study, severe CVID patients had significantly lower percentages of NK cells compared to moderate patients. However, no significant difference was found between all patients and healthy controls (Table 4). NK cell deficiency in severe CVID cases might amplify the defective functioning of other compartments of the immune system, further compromising tumor surveillance and protection against viral infection (1).

CD28, the major co-stimulatory ligand for CD80/86, has been reported to be present on the surface of human NK cells (27). CD28 costimulation has been shown to induce optimal proliferation of murine NK cells and enhance IFN-γ secretion, and the CD28 pathway has been shown to be involved in cytotoxic killing of tumor cells (10). In addition, CD28-deficient NK cells were shown to have markedly reduced ability to lyse syngeneic tumor cells (10). However, some other studies have raised doubts on the expression of CD28 by human NK cells using a series of mAbs recognizing CD28 only on T cells (10). Therefore, the role of the CD28 co-stimulatory pathway in influencing the nature of NK cell responses remains controversial. In our study, on CD3^−^CD16^+^CD56^+^ NK cells of CVID patients, the percentages of those with CD28^+^ were significantly higher (*p*: 0.008) than controls while CD28^−^ NK cells were significantly decreased in CVID cases (*p*: 0.006) (Table 4). This finding may be related to a compensatory mechanism for protection against viral infections and also for prevention of developing tumors such as lymphoma. We had only two CVID cases with lymphoma and we could not find any relationship between lymphoma formation and percentage of CD28-deficient NK cells.

Natural killer cell-mediated cytotoxicity is believed to play an important role in immune surveillance against cancer (28). The possible role of NK cell-mediated immunity and the development of malignant disease in humans is one of the popular fields of investigation in recent years. It is still not clear whether CVID patients with or without lymphoma present depressed or increased NK cytotoxicity. Orange et al. (29) evaluated three patients with NEMO (NF-κB essential modifier) mutations and HED-ID (hypohidrotic ectodermal dysplasia with immune deficiency). These patients had normal percentages of peripheral blood NK cells, but impaired NK cell cytotoxic activity. The authors reported that NEMO participates in signaling pathways leading to NK cell cytotoxicity and that IL-2 can activate NF-κB and partially overcome the NK cell defect in patients with NEMO mutation (29). On the other hand, Liu et al. (30) identified a novel NF-κB2 mutation in early-onset CVID and reported that NF-κB2 gene mutations are one of the causative agents for this heterogeneous disease (29). In our study, CVID patients had strikingly increased NK cell cytoxicity, CD28^+^ NK cell ratio in comparison with healthy controls. This may be the result of repeated activation in patients due to recurrent infections and autoimmunity, showing enhanced lytic activity, thus suggesting an intact NK cell function in CVID patients.

Because of the important interactions of B cells with NKT cells, we measured their frequencies in CVID patients and compared them with healthy controls. CVID represents a spectrum of diseases, and different genetic causes might lead to differences in NKT cell expression. There are very limited results to assess NKT cell frequency in CVID patients. In Carvalho’s study with CVID patients, CD4^+^NKT cells were at higher frequencies and CD8^+^NKT cells were at lower frequencies (9). In the same study, higher percentages of NKT cells expressed CCR5 in CVID patients when compared with healthy controls. In a study by Trujillo et al. (2), CD1d-restricted TCR invariant natural killer cells (iNKT) cells were significantly decreased in the peripheral blood of most of the CVID patients. In our study, CD3^+^CD16^+^CD56^+^ cell percentages did not show any difference between patient and control groups or between severe and moderate groups. Neither CD28^+^ nor CD28^−^ cell percentages on NKT cells showed difference between the two groups (Table 6). Further studies are needed to clarify whether altered absolute numbers or functions of NKT cells in CVID patients could play a role for the impaired B cell function.

Blood monocyte and neutrophil leukocyte phagocytic functions can be evaluated by chemotaxis, phagocytosis, and superoxide anion production. We addressed whether CVID is associated with abnormalities in polymorphonuclear neutrophils as they constitute an important compartment of innate immunity and play a key role in host defense against invading microorganisms. We used flow cytometry to examine PMN functional abnormalities in CVID patients, using whole blood. Four CVID patients and one in the control group showed decreased migration capacity. Percentage of migrated granulocytes was significantly decreased in CVID patients compared to controls (Table 7). In addition, oxidative burst activity was found to be decreased in six patients (30%), but no significant difference was found between disease and control groups regarding oxidative burst activity. Amoras et al. (31) evaluated these functions in 9 CVID, 8 XLA (X-linked agammaglobulinemia), and 17 normal subjects. Phagocytosis was found to be significantly decreased in CVID and XLA patients and chemotaxis of monocytes was reduced in XLA patients. Superoxide anion production, however, did not differ between CVID, XLA, and the control groups (31). Casulli et al. (32) demonstrated that PMN from CVID patients exhibited a decrease in degranulation, phagocytosis, and reactive oxygen species production. Badolato (33) explained the mechanism of this aberration and reported that lower levels of CXCR5 and CCR7 are associated with reduced migration of these cells to CXCL13 and CCL21/CCL19, respectively, thereby preventing their homing. It is proposed that impaired chemotaxis and phagocytosis of phagocytic cells may be characteristics of the innate immune system in CVID patients, providing a new direction for the pathogenesis of this immunodeficiency. However, our current results did not confirm this comment.

It is very important for lymphocytes to home to lymph nodes. Upon cell activation, L-selectin is rapidly shed and a tighter adhesion between leukocytes and endothelial cells is mediated by integrins (e.g., lymphocyte function-association antigen-1, LFA-1; CD11a, CD18) (5). Since adhesion molecules are important in various inflammatory and immunological responses, we wanted to examine whether CVID patients showed any disturbances in the expression of CD11a and CD18 on lymphocytes and neutrophils. CD11a percentages on lymphocytes and neutrophils showed significant decrease in patients in relation to control groups. CD18 percentages did not differ between groups but CD18 MFI was significantly increased in patients suggesting an activated stage of leukocytes (Table 8) (Figure 2). The changes we have observed in these adhesion molecules may have clinical implications in CVID. In our study, decreased neutrophilic CD11a expression was associated with the presence of celiac-like disease and bronchiectasis. The altered expression of these molecules on leukocyte subsets may possibly influence the interactions between these cells, which may impair the immune response of patients.

This is the first study examining migratory function of granulocytes, oxidative burst activity of phagocytic cells, surface integrin expressions on neutrophils and lymphocytes, NK cell numbers and cytotoxic activity, NKT cell numbers, and lymphocyte subsets such as CD8^+^CD28^+^, CD4^+^CTLA-4^+^ cells in the same CVID patient group. As a result, CD3^+^CD8^+^ T cytotoxic cells were found to be elevated in CVID patients, but CD3^+^CD8^+^CD28^+^ or CD3^+^CD8^+^CD28^−^ cells did not show any difference. Severe CVID patients had decreased percentages of NK cells and increased NK cell cytotoxicity, suggesting repeated activation of this system due to infections. Furthermore, CD3^−^CD16^+^CD56^+^CD28^+^ cells of CVID patients were elevated while percentage of CD28^−^ NK cells was decreased. Neutrophil migration percentages were lower but oxidative burst activity was not affected in the patient group. CD18 expression on leukocytes was increased suggesting an activated stage. CD11a expressions on these cells were depressed.

One of the limitations of our study is the small number of patients divided in relation to their clinical characteristics, which may reduce the power of statistical analyses performed. Another limitation is the fact that all of our patients receive IVIg periodically. Although some of the values did not differ between patient and healthy controls, it would be best to have the opportunity to test these patients before treatment, also. As reported in previous studies, IVIgs modulate the ability of the innate immunity to trigger T cell activation, influence NK cell-dependent tumor surveillance by protein–glycan interactions and modulate expression of adhesion molecule on cell surfaces (34–36).

Generation and routine use of a functional innate test system will help clinicians better correlate clinical phenotypes and provide more accurate molecular diagnostic approach for CVID patients.

## Conflict of Interest Statement

The authors declare that the research was conducted in the absence of any commercial or financial relationships that could be construed as a potential conflict of interest.
